# Exact Interior Reconstruction from Truncated Limited-Angle Projection Data

**DOI:** 10.1155/2008/427989

**Published:** 2008-05-06

**Authors:** Yangbo Ye, Hengyong Yu, Ge Wang

**Affiliations:** ^1^Department of Mathematics, University of Iowa, Iowa City, IA 52242, USA; ^2^CT Laboratory, Biomedical Imaging Division, VT-WFU School of Biomedical Engineering, Virginia Tech, Blacksburg, VA 24061, USA

## Abstract

Using filtered backprojection (FBP) and an analytic continuation approach, we prove that exact interior reconstruction is possible and unique from truncated limited-angle projection data, if we assume a prior knowledge on a subregion or subvolume within an object to be reconstructed. Our results show that (i) the interior region-of-interest (ROI) problem and interior volume-of-interest (VOI) problem can be exactly reconstructed from a limited-angle scan of the ROI/VOI and a 180 degree PI-scan of the subregion or subvolume and (ii) the whole object function can be exactly reconstructed from nontruncated projections from a limited-angle scan. These results improve the classical theory of Hamaker et al. (1980).

## 1. INTRODUCTION

The importance of performing exact image reconstruction from the minimum amount of data has been recognized for a long time. The first landmark achievement is the well-known fan-beam half-scan formula [1]. A recent milestone is the two-step Hilbert transform method developed by Noo et al. [2] in 2004 In their framework, an object image on a PI-line/chord can be exactly reconstructed if the intersection between the chord and the object is completely covered by a field of view (FOV). In 2006, Defrise et al. [3] proposed an enhanced data completeness condition that the image on a chord in the FOV can be exactly reconstructed if one end of the chord in the object is covered by the FOV. Inspired by the tremendous biomedical implications including local cardiac CT at minimum dose, local dental CT with high accuracy, CT guided procedures, and nano-CT using analytic continuation we recently proved that the interior problem can be exactly and stably solved if a subregion in an ROI/VOI in the FOV is known [4–7] from fan-beam/cone-beam projection datasets, while the conventional wisdom that the interior problem does not have a unique solution [8] remains correct.

Using the analytic continuation technique, here we further extend our exact interior reconstruction results to the case of a truncated limited-angle scan. The paper is organized as follows. In the next section, we summarize the relevant notations and key theorem. In the third section, we prove our theorem in the filtering backprojection (FBP) framework. In the fourth section, we will discuss relevant ideas and conclude the paper.

## 2. NOTATIONS AND KEY THEOREM

The basic setting of our previous work is cone-beam scanning along a general smooth trajectory

(1) Γ={ ρ(s) ∣ s∈ℝ}.

**Figure 1:**
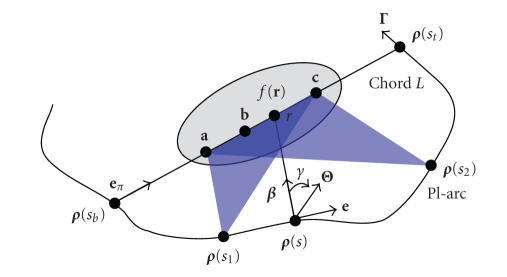
Basic setting for exact 3D interior reconstruction from truncated limited-angle datasets.

As shown in Figure 1, a generalized PI-line of **r** ∈ ℝ^3^ is defined as the line through **r** and across the scanning trajectory at two points ***ρ*** ( *s_b_* ) and ***ρ*** (*s_t_*) on **Γ** with *s_b_* < *s_t_*, where *s_b_* = *s_b_* (**r**) and *s_t_* = *s_t_* (**r**) are the parameter values corresponding to these two points. At the same time, the generalized PI-segment (also referred to as a chord) *L* is defined as the segment of the PI-line between ***ρ*** ( *s_b_* ) and ***ρ*** (*s_t_*), the PI-arc the part of the trajectory between ***ρ*** (*s_b_*) and ***ρ*** ( *s_t_* ), and the PI-interval [ *s_b_* , *s_t_* ]. Suppose that an object function *f*(**r**) is constrained in a compact support **Ω** ⊂ ℝ^3^. For any unit vector ***β***, let us define a cone-beam projection of *f*(**r**) from a source point ***ρ*** (*s*) on the trajectory **Γ** by

(2) $$D_{f}(\rho (s),\beta ):=\int_{0}^{\infty }f(\rho (s)+t\beta )dt.$$

Then we define a unit vector ***β*** (*s*, **r**) as the one pointing to **r** ∈ *L* from ***ρ*** (*s*) on the trajectory:

(3) $$\beta (r,s):=\frac{r-\rho (s)}{\left|r-\rho (s)\right|}.$$

We also need a unit vector along the chord:

(4) $$e_{\pi }:=\frac{\rho (s_{t})-\rho (s_{b})}{\left|\rho (s_{t})-\rho (s_{b})\right|}.$$

Note that the unit vector **e**_π_ is the same for all **r** ∈ *L*. Our main finding can be summarized as the following theorem.

**Theorem 1.**

*Assume that there are three points* **a**, **b**, **c** *on the chord L with* **b** *situating between* **a** *and* **c**. *Suppose that (i) projection data* *D_f_* (***ρ*** (*s*), ***β*** (**r**, *s*)) *are known and D_f_* (***ρ*** (*s*), −***β*** (**r**, *s*)) ≡ 0, *both for any s* ∈ [*s_b_*, *s_t_*] *and for any* **r** *on the line-segment* $\bar{ab}$ *and a small neighborhood;* (ii) *projection data D_f_* (***ρ*** (*s*), ***β*** (**r**, *s*)) *are known and D_f_* (***ρ*** (*s*), −***β*** (**r**, *s*)) ≡ 0, *both for any s* ∈ [*s*_1_, *s*_2_] *with s_b_* < *s*_1_ < *s*_2_ < *s_t_ and for any* **r** *on the line-segment* $\bar{bc}$ *and a small neighborhood; and* (iii) *f*(**r**) *is known on the line-segment* $\bar{ab}$ . *Then the function* *f*(**r**) *can be exactly reconstructed on the line-segment* $\bar{bc}$ .

Let us remark on the conditions for Theorem 1 Our conditions (i) and (ii) imply that the cone-beam projection data are both longitudinally and transversely truncated but the derivative (∂/∂*q*)*D_f_* (***ρ*** (*q*), ***β*** (**r**, s))|_*q*=*s*_ is available for any *s* ∈ [*s_b_*, *s_t_*] and any **r** on line-segment $\bar{ab}$ , which we define as data from a PI-scan, and for any *s* ∈ [*s*_1_, *s*_2_] and any **r** on line-segment $\bar{bc}$ . Because the amount of data (∂/∂*q*)*D_f_* (***ρ*** (*q*), ***β*** (**r**, s))|_*q*=*s*_ is less than a PI-scan for **r** on line-segment $\bar{bc}$ , we have the limited-angle problem. Our condition (iii) demands a priori information for the exact interior reconstruction. We may also assume that the known data are on subintervals of the line-segment $\bar{ab}$ . In practice, the function *f*(**r**) can be often known inside a subregion of the VOI, such as air around a tooth, water in a chamber, or calibrated metal in a semiconductor.

## 3. PROOF OF THEOREM 1

Based on Katsevich's work [9, 10], early 2005 Ye and Wang proved a generalized FBP method that performs filtering along a generalized PI-line direction [11]. They also derived a generalized filtering condition for exact FBP reconstruction [11], which is special case of Katsevich's general weighting condition [10]. For an arbitrary smooth scanning curve ***ρ*** (*s*) on the generalized PI-interval [*s_b_*, *s_t_*] and any point **r** on the chord *L* from ***ρ*** (*s_b_*) to ***ρ*** (*s_t_*), the exact FBP reconstruction formula can be expressed as [11] follows:

(5) $$\begin{array}{ll} f(r) & =-\frac{1}{2\pi^{2}}\int_{s_{b}}^{s_{t}}\frac{ds}{\left|r-\rho (s)\right|}\\ & \;\times PV\int_{0}^{2\pi }\frac{\partial }{\partial q}{D_{f}(\rho (q),\Theta (s,r,\gamma ))|}_{q=s}\frac{d\gamma }{\sin\gamma } \end{array}$$

where “PV” represents a principal value integral, and **Θ**(*s*, **r**, *γ*) the filtering direction which is taken in the PI-segment direction and defined as cos*γ* ***β*** + sin *γ* **e** with the unit directions ***β*** = ***β*** (**r**, *s*) and **e** = (**e**_π_ − (**e**_π_· ***β***) ***β***)/(|**e**_π_ − (**e**_π_· ***β***) ***β*** |), *that is*, **Θ**(*s*, **r**, *γ*) supposes a clockwise rotation in the plane determined by *L* and ***β*** (**r**, *s*), centered at ***ρ*** (*s*) with **Θ**(*s*, **r**, 0) = ***β*** (**r**, *s*) (see Figure 1).

**Figure 2:**
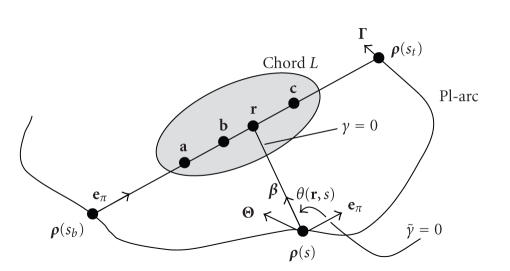
Variable change from *γ* to $\overset{˜}{\gamma }$ .

For a fixed point ***ρ*** (*s*), the filtering plane remains unchanged for all **r** ∈ *L*. Following the same steps as in our previous work [6], we can change the variable *γ* to $\tilde{\gamma }$ so that the direction for $\tilde{\gamma }=0$ now points to the direction **e**_π_, and the filtering direction is still specified clockwise (see Figure 2). Let *θ*(**r**, *s*) denote the angle from **e**_π_ ( $\tilde{\gamma }=0$ ) to ***β*** (**r**, *s*). Then (5) can be rewritten as

(6) $$\begin{array}{ll} f(r) & =-\frac{1}{2\pi^{2}}\int_{s_{b}}^{s_{t}}\frac{ds}{\left|r-\rho (s)\right|}\text{P}\text{V}\int_{-\pi }^{\pi }\frac{\partial }{\partial q}\\ & \;\times {D_{f}(\rho (q),\Theta (s,\overset{˜}{\gamma }\;))|}_{q=s}\frac{d\overset{˜}{\gamma }}{\sin(\overset{˜}{\gamma }-\theta (r,s))}. \end{array}$$

Note that **Θ**(*s*, **r**, *γ*) now is changed to $\Theta (s,\tilde{\gamma })$ which is independent of **r** ∈ *L*, and the value of *θ*(**r**, *s*) is negative.

From (6) with PI-line filtering, we have

(7) $$\begin{array}{ll} f(r) & =-\frac{1}{2\pi^{2}}\int_{s_{1}}^{s_{2}}\frac{ds}{\left|r-\rho (s)\right|}\text{P}\text{V}\int_{\theta (a,s)}^{\theta (c,s)}\frac{\partial }{\partial q}\\ & \;\times {D_{f}(\rho (q),\Theta (s,\overset{˜}{\gamma }\;))\;|}_{q=s}\frac{d\overset{˜}{\gamma }}{\sin(\overset{˜}{\gamma }-\theta (r,s))} \end{array}$$

(8) $$\begin{array}{ll} & \;-\frac{1}{2\pi^{2}}\int_{s_{b}}^{s_{t}}\frac{ds}{\left|r-\rho (s)\right|}\text{P}\text{V}\left(\int_{-\pi }^{\theta (a,s)}+\int_{\theta (c,s)}^{\pi }\right)\\ & \;\times \frac{\partial }{\partial q}{D_{f}(\rho (q),\Theta (s,\overset{˜}{\gamma }))\;|}_{q=s}\frac{d\overset{˜}{\gamma }}{\sin(\overset{˜}{\gamma }-\theta (r,s))} \end{array}$$

(9) $$\begin{array}{ll} & \;-\frac{1}{2\pi^{2}}\left(\int_{s_{b}}^{s_{1}}+\int_{s_{2}}^{s_{t}}\right)\frac{ds}{\left|r-\rho (s)\right|}\text{P}\text{V}\int_{\theta (a,s)}^{\theta (b,s)}\frac{\partial }{\partial q}\\ & \;\times {D_{f}(\rho (q),\Theta (s,\overset{˜}{\gamma }\;))\;|}_{q=s}\frac{d\overset{˜}{\gamma }}{\sin(\overset{˜}{\gamma }-\theta (r,s))} \end{array}$$

(10) $$\begin{array}{ll} & \;-\frac{1}{2\pi^{2}}\left(\int_{s_{b}}^{s_{1}}+\int_{s_{2}}^{s_{t}}\right)\frac{ds}{\left|r-\rho (s)\right|}\text{P}\text{V}\int_{\theta (b,s)}^{\theta (c,s)}\frac{\partial }{\partial q}\\ & \;\times {D_{f}(\rho (q),\Theta (s,\overset{˜}{\gamma }\;))\;|}_{q=s}\frac{d\overset{˜}{\gamma }}{\sin(\overset{˜}{\gamma }-\theta (r,s))}. \end{array}$$

Here (7) and (9) are known for the given truncated projection data from our conditions (i) and (ii). As in [6], we can rewritten (8) as

(11) $$\begin{array}{ll} & \;-\frac{1}{2\pi^{2}}\int_{s_{b}}^{s_{t}}ds\text{P}\text{V}(\int_{-\pi }^{\theta (a,s)}+\int_{\theta (c,s)}^{\pi })\frac{\partial }{\partial q}{D_{f}(\rho (q),\Theta (s,\overset{˜}{\gamma }\;))|}_{q=s}\\ & \;\;\times \frac{d\overset{˜}{\gamma }}{\sin\;\overset{˜}{\gamma }(r\;-\;r_{p}(s))\;+\;\cos\;\overset{˜}{\gamma }\;\left|\;r_{p}(s)\;-\;\rho (s)\right|}. \end{array}$$

**Figure 3:**
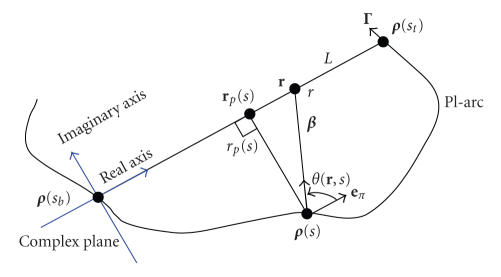
Complex coordinate system for the analytic continuity.

Here **r**_*p*_(*s*) is the point on *L* such that **r**_*p*_(*s*) − ***ρ*** (*s*) is perpendicular to *L*. We set up a complex plane *ℂ* with its origin at ***ρ*** (*s_b_*) and real axis from ***ρ*** (*s_b_*) to ***ρ*** (*s_t_*) (see Figure 3). Using this complex plane, we rename ***ρ*** (*s_b_*) as *O*, **a** as *a*, **r** as *r*, **r**_*p*_(*s*) as **r**_*p*_(*s*), and so on, on the real axis. We note that when *r* ∈ (*a*, *c*), the PV integrals in (11) are actually ordinary integrals and hence integrals of Cauchy’s type. By the Cauchy integral theorem, (11) and (8) represent an analytic function on the complex plane *ℂ* with cuts (−∞, *a*] and [*c*, +∞) on the real axis.

Now we return to (10) and rewrite it as

(12) $$\begin{array}{ll} f_{11}(r) & =-\frac{1}{2\pi^{2}}\left(\int_{s_{b}}^{s_{1}}+\int_{s_{2}}^{s_{t}}\right)ds\\ & \;\times {PV\int }_{\theta (b,s)}^{\theta (c,s)}\frac{\partial }{\partial q}{D_{f}(\rho (q),\Theta (s,\overset{˜}{\gamma }\;))|}_{q=s}\\ & \;\times \frac{d\overset{˜}{\gamma }}{\sin\;\overset{˜}{\gamma }(r-r_{p}(s))+\cos\;\overset{˜}{\gamma }\left|r_{p}(s)-\rho (s)\right|}. \end{array}$$

Equation (12) defines an analytic function *f*_11_(*r*) in the complex plane with a cut [*b*, ∞) along the real axis, because for *r* ∉ [*b*, *c*], the inner integral in (12) is an ordinary integral and an integral of Cauchy type. If *r* ∈ (*b*, *c*), *f*_11_(*r*) is not analytic. The values of *f*_11_ on (*b*, *c*), however, can still be determined uniquely by the analytic function *f*_11_(*r*) on *ℂ* \ [*b*, *c*]. Indeed, for *r* ∈ (*b*, *c*),

(13) $$f_{11}\left(r\right)=\frac{1}{2}\underset{\underset{\text{Im }z>0}{z\to r}}{\lim}\;f_{11}\left(z\right)+\frac{1}{2}\underset{\underset{\text{Im }z<0}{z\to r}}{\lim}\;f_{11}\left(z\right).$$

Back to (6), now we have

(14) f(r)=f(r)=(8)+(9)+(10)+(13).

Recall that (7) and (9) are known for any **r** from our projection data, (8) is an analytic function on the complex plane with cuts (−∞, *a*] and [*c*, +∞), and (12) is a single-valued analytic function on the complex plane *ℂ* with cuts [*b*, *c*] along the real axis. Therefore, (8) + (12) is an analytic function on *ℂ*/(−∞, *a*] ∪ [*b*, ∞). Since *f*(*r*) is known on (*a*, *b*), (8) + (12) is known on (*a*, *b*). This uniquely determines the analytic function (8) + (12). Denote this analytic function as by *h*(*z*) for *z* ∈ *ℂ*/(−∞, *a*] ∪ [*b*, ∞). In order to reconstruct *f*(*r*) for *r* ∈ (*b*, *c*), however, we need to know *h*(*r*) for *r* ∈ (*b*, *c*). This can be done using (13). Equation (13) obviously holds for (8) too, because it is analytic on (*b*, *c*). Consequently,

(15) $$h\left(r\right)=\frac{1}{2}\underset{\underset{\text{Im }z>0}{z\to r}}{\lim}\;h\left(z\right)+\frac{1}{2}\underset{\underset{\text{Im }z<0}{z\to r}}{\lim}\;h\left(z\right)$$

Using (15) to compute the value of (8) + (12) at *r* ∈ (*b*, *c*), and using the known values of (7) and (9) at *r* ∈ (*b*, *c*), we finally can reconstruct *f*(*r*) on (*b*, *c*) exactly.

## 4. DISCUSSIONS AND CONCLUSION

Because the exact interior reconstruction is unique from truncated limited-angle data according to Theorem 1, there are many interesting applications we should work on for exact reconstruction, including but not limited to traditional limited-angle tomography, circular cone-beam tomography, and reconstruction of a flat or plate-like object from data collected along a planer curve below or above the flat object [12]. Clearly, for practical applications we may stabilize the exact reconstruction process using various means such as penalty measures and knowledge-based constraints. We emphasize that other types of knowledge may also be incorporated in our exact interior reconstruction; for example, a digital atlas of the family of object under study As long as we use sufficient constraints, the theoretically exact reconstruction nature will surely be enhanced by numerical stability. We also acknowledge that the resolution or image quality with the truncated limited-angle scan could be affected by the scanning angle, sampling rate, detector resolution, and so on. Major efforts on research analysis, numerical simulation, and physical experiment are needed along this more promising direction.

**Figure 4:**
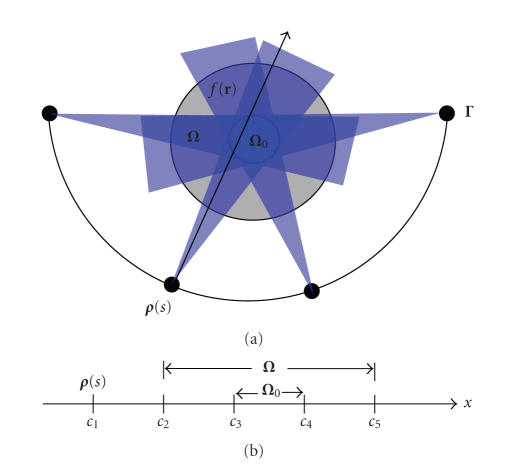
(a) Illustration of the subregion/volume half-scan ROI problem; (b) the 1D coordinate system along the X-ray path indicated in (a).

As an inspiring case, let us consider the 2D ROI-focused scan illustrated in Figure 4(a) Assume that there is a subregion **Ω**_0_ (white region) inside the compact support **Ω** that is half-scanned; namely, **Ω**_0_ satisfies the half-scan reconstruction condition if *f*(**r**) ≡ 0 for **r** ∈ (**Ω** − **Ω**_0_) in the gray region. Although the projection data is generally truncated in this setting, it can still be scanned by a limited-angle for any **r** ∈ (**Ω** − **Ω**_0_). Our theorem implies that we can exactly reconstruct the object function *f*(**r**) on the whole support **Ω** if we have known the object function *f*(**r**) in **Ω**_0_. Based on our previous results [4–6], the prior information can be reduced to a measurable subregion in **Ω**_0_. This result can also be proved in the backprojection filtration (BPF) framework. Let us consider an X-ray path from any source ***ρ*** (*s*) on the scanning trajectory and going through both **Ω** and **Ω**_0_. We can set up a 1D coordinate system along this X-ray path (see Figure 4(b)). Denote the 1D coordinate of ***ρ*** (*s*) as *c*_1_, the coordinates of the intersections with **Ω** as *c*_2_ and *c*_5_, the coordinates of the intersections with **Ω**_0_ as *c*_3_ and *c*_4_, and *c*_1_ < *c*_2_ < *c*_3_ < *c*_4_ < *c*_5_. In this 1D case, *f*(*x*) is supported on [*c*_2_, *c*_5_] and *f*(*x*) is known on (*c*_3_, *c*_4_). According to the results of Pack et al. [13], the 1D Hilbert transform *g*(*x*) of *f*(*x*) can be exactly obtained on the interval [*c*_3_, *c*_4_]. Based on the inverse Hilbert Transform [2, 14], we have

(16) $$\begin{array}{ll} & \sqrt{\left(c_{5}-x)(x-c_{2}\right)}f(x)\\ & \;\;\begin{array}{c} =\int_{c_{3}}^{c_{4}}\frac{\sqrt{\left(c_{5}-\overset{˜}{x}\;)(\overset{˜}{x}-c_{2}\right)}g(\overset{˜}{x})}{\pi (\overset{˜}{x}-x)}d\overset{˜}{x}+\frac{1}{\pi }\int_{c_{2}}^{c_{5}}f(\overset{˜}{x}\;)d\overset{˜}{x} \end{array} \end{array}$$

(17) $$\begin{array}{ll} & \;\;\;+\left(\int_{c_{2}}^{c_{3}}+\int_{c_{4}}^{c_{5}}\right)\frac{d\overset{˜}{x}\sqrt{\left(c_{5}-\overset{˜}{x}\;)(\overset{˜}{x}-c_{2}\right)}g(\overset{˜}{x}\;)}{\pi (\overset{˜}{x}-x)} \end{array}.$$

Note that (16) is known for any *x* ∈ (*c*_2_, *c*_5_), (17) is an analytic function with cuts on (−∞, *c*_3_] and [*c*_4_, ∞). Because *f*(*x*) is known on (*c*_3_, *c*_4_), (17) is also known on (*c*_3_, *c*_4_). By the same argument as for (13), we can extend the values of (17) from (*c*_3_, *c*_4_) to [*c*_2_, *c*_5_]. Hence *f*(*x*) can be exactly reconstructed on the whole interval [*c*_2_, *c*_5_].

**Figure 5:**
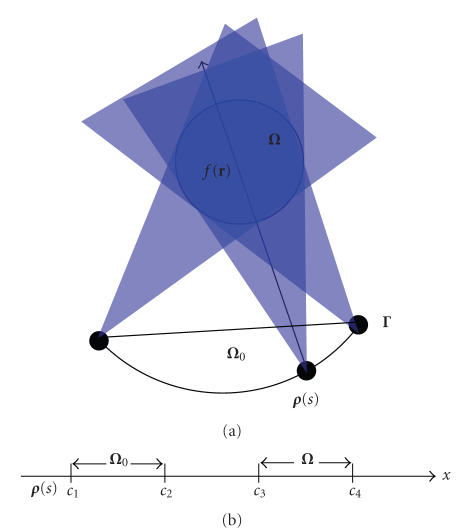
(a) Illustration of nontruncated limited-angle scanning problem; (b) the 1D coordinate system along the X-ray path indicated in (a).

Furthermore, let us revisit the so-called nontruncated limited-angle scanning problem. For clarity, we only consider the 2D case as illustrated in Figure 5(a). We assume that it can form a measurable region **Ω**_0_ by connecting two endpoints of the limited-angle scanning trajectory. Again, let us consider an X-ray path from any source ***ρ*** (*s*) on the scanning trajectory and through the compact support **Ω**. We can set up a 1D coordinate system along this X-ray path. Denote the 1D coordinate of ***ρ*** (*s*) as *c*_1_, the coordinates of the other intersection with **Ω**_0_ as *c*_2_, the coordinates of the intersections with **Ω** as *c*_3_ and *c*_4_, with *c*_1_ < *c*_2_ < *c*_3_ < *c*_4_. In this 1D case, *f*(*x*) is supported on [*c*_3_, *c*_4_] and *f*(*x*) = 0 for *x* ∈ (*c*_1_, *c*_2_). According to the results of Pack et al. [13], the 1D Hilbert transform *g*(*x*) of *f*(*x*) can be exactly obtained on the interval [*c*_1_, *c*_2_]. Based on the inverse Hilbert Transform [2, 14], we have

(18) $$\begin{array}{ll} & \sqrt{\left(c_{4}-x)(x-c_{1}\right)}f(x)\\ & \;\;=\int_{c_{1}}^{c_{2}}\frac{\sqrt{\left(c_{4}-\overset{˜}{x})(\overset{˜}{x}-c_{1}\right)}g(\overset{˜}{x})}{\pi (\overset{˜}{x}-x)}d\overset{˜}{x}+\frac{1}{\pi }\int_{c_{3}}^{c_{4}}f(\overset{˜}{x})d\overset{˜}{x} \end{array}$$

(19) $$\begin{array}{ll} & \;\;\;+\int_{c_{2}}^{c_{4}}\frac{d\overset{˜}{x}\sqrt{\left(c_{4}-\overset{˜}{x})(\overset{˜}{x}-c_{1}\right)}g(\overset{˜}{x})}{\pi (\overset{˜}{x}-x)}. \end{array}$$

While (18) is known for *x* ∈ [*c*_1_, *c*_4_], (19) is an analytic function with a cut on [*c*_2_, *c*_4_]. Because *f*(*x*) is known on (*c*_1_, *c*_2_), (19) is also known on (*c*_1_, *c*_2_). Following the same argument as for (13), we can extend the values of (19) from (*c*_1_, *c*_2_) to [*c*_2_, *c*_4_]. Thus, *f*(*x*) can be exactly reconstructed on [*c*_3_, *c*_4_]. This result is consistent with Theorem 5.1 by Hamaker et al. in [15].

Although our work has been done within the X-ray CT framework, our results can be directly applied to other tomographic modalities that share similar imaging models such as MRI, ultrasound imaging, PET, and SPECT. By similarity between imaging models, we underline that the exponential Radon transform is a particular attractive area since a generalized Hilbert transform theory has been reported for exact reconstruction from transversely truncated data [16, 17]. Clearly, extensions into higher dimensions and time-varying cases are theoretically possible as well. In all these cases, iterative algorithms can always be adapted or developed to produce optimal results, which can be stabilized or regularized subject to various constraints [18–23].

In conclusion, we have proved that the exact interior reconstruction is theoretically solvable. Theorem 1 and key techniques in its proof have numerous practical implications. Hopefully, our results have opened a new direction to advance the local reconstruction area. We are actively working on exciting possibilities discussed above.
